# Clinical Treatment Efficacy of Total Thyroidectomy Combined with Radioactive Iodine on Treatment of Thyroid Cancer and Its Effect on the Quality of Life of Patients

**Published:** 2019-08

**Authors:** Yong YANG, Yanqin JIAO, Jieqing YU, Chenxiu WANG

**Affiliations:** 1. Department of Vascular Mammary Surgery (Thyroid and Hernia), Jiangxi Provincial People’s Hospital, Nanchang University, Nanchang, 330006, P.R. China; 2. Department of Nursing, Jiangxi Health Vocational College, Nanchang, 330052, P.R. China; 3. Department of Otolaryngology Head and Neck Surgery, The First Affiliated Hospital of Nanchang University, Nanchang, 330006, P.R. China; 4. Department of Endocrine, Jiangxi Provincial People’s Hospital, Nanchang University, Nanchang, 330006, P.R. China

**Keywords:** Total thyroidectomy, Radioactive iodine therapy, Thyroid cancer, Quality of life

## Abstract

**Background:**

To investigate the treatment efficacy of radioactive iodine therapy on patients after total thyroidectomy and its effect on the quality of life.

**Methods:**

A retrospective analysis of clinical data of 120 thyroid cancer patients admitted to Jiangxi Provincial People’s Hospital Affiliated to Nanchang University, Nanchang, China from February 2014 to February 2017 was performed. According to different treatment methods, they were divided into observation group of 62 cases and control group of 58 cases. Both groups were treated with total thyroidectomy. The control group was treated with anti-infection and prevention of complications after operation, the observation group with radioactive iodine therapy. Treatment efficacy, quality of life score, recurrent laryngeal nerve injury and postoperative survival rate were compared between the two groups.

**Results:**

The total effective rate of treatment in the test group was 98.39%, significantly higher than 72.41% in the control group, with a statistically significant difference (*P*<0.05). Compared with the control group, the fatigue score of the test group was lower, but the score in the area of emotion function and the overall health status score were higher, with a statistically significant difference (*P*<0.05). There was no significant difference in the recurrent laryngeal nerve injury between the two groups of patients. The postoperative survival rate of the test group of patients was 96.77%, significantly higher than 86.21% of the control group.

**Conclusion:**

The effect of radioactive iodine therapy after total thyroidectomy is remarkable, which can significantly improve the clinical treatment efficacy and postoperative quality of life of patients, worthy of clinical application.

## Introduction

Thyroid cancer is a common endocrine malignant tumor in clinic. In recent years, due to changes in the living environment, its incidence has been increasing year by year, with females higher than males (1, 2). Most of the tumor cells of thyroid cancer are derived from follicular epithelial cells, according to pathological type, which are classified into papillary adenocarcinoma, follicular adenocarcinoma, medullary carcinoma and undifferentiated carcinoma. Among them, the incidence of papillary adenocarcinoma is the highest, up to 80% (3). Usually without obvious symptoms in early stage, thyroid cancer should be treated surgically if diagnosed (4).

Conducive to clearing the lesion and accurately staging the tumor, total thyroidectomy has become an ideal clinical treatment option, easy to use postoperative individualized treatment. However, only a single surgical treatment cannot significantly improve the postoperative metastasis and recurrence rate of cancer (5). It has a certain negative impact on the quality of life of patients in the short term (6). At present, with the increasing living standards of people, the survival period is not the only criterion for evaluating the quality of cancer treatment method. More and more attention has been paid to the quality of life of cancer patients after treatment (7). As an internal radiotherapy, in order to improve the quality of life and clinical treatment efficacy of patients after operation, radioactive iodine therapy has been clinically advocated to treat thyroid cancer patients after total thyroidectomy (8). Radioactive iodine therapy is applicable for patients with residual glandular tissue after surgery or after recurrence or metastases that cannot be surgically removed after surgery, and radioactive iodine scans have been found in patients with radioactive iodine (9). It can allow the iodine pump to actively operate, so that iodide ions can accelerate into the thyroid cells. In addition, the lesion can be found, metastasized and removed by it, enhancing operative efficacy to prevent the recurrence of cancer (10).

Therefore, in this study, the clinical treatment efficacy of total thyroidectomy combined with radioactive iodine in the treatment of thyroid cancer and its impact on the quality of life of patients were explored, in order to provide a better solution for the treatment of thyroid cancer.

## Materials and Methods

### General information

A retrospective analysis of 120 thyroid cancer patients undergoing total thyroidectomy admitted to Jiangxi Provincial People’s Hospital Affiliated to Nanchang University, China from February 2014 to February 2017 was performed, including 47 males and 73 females, with an average age of (45.12±9.11) yr old. According to the pathological type, there were 87 cases of papillary adenocarcinoma, 33 cases of follicular adenocarcinoma. TNM staging: 67 cases in stage I, 36 cases in stage II and 17 cases in stage III. Among them, 62 cases treated with radioactive iodine therapy after operations were in the test group, 58 cases with conventional anti-infection and prevention of complications in the control group. There was no difference between two groups on sex, age, BMI, malignant type and TNM staging (Table 1).

**Table 1:** Comparison of general data between two groups of patients [n(%)]

***Factors***	***Test group (n=62)***	***Control group (n=58)***	***X^2^***	***P***
Sex			0.011	0.916
Male	24 (38.71)	23 (39.66)		
Female	38 (61.29)	35 (60.34)		
Age (yr)			0.013	0.909
≤40	22 (35.48)	20 (34.48)		
>40	40 (64.52)	38 (65.52)		
BMI (kg/m^2)^			0.001	0.971
≤22	28 (45.16)	26 (44.83)		
>22	34 (54.84)	32 (55.17)		
Malignant type			0. 151	0. 698
Papillary carcinoma	44(70.97)	43(74.14)		
Follicular carcinoma	18(29.03)	15(25.86)		
TNM staging			0.171	0.918
Stage I	35 (56.45)	32 (55.17)		
Stage II	19 (30.65)	17 (29.31)		
Stage III	8 (12.90)	9 (15.52)		
Surgical method			0.247	0.884
Unilateral total resection plus isthmus resection plus contra-lateral subtotal resection	41 (66.13)	38 (65.52)		
Bilateral total resection	10 (16.13)	8 (13.79)		
Unilateral total resection plus isthmus resection	11 (17.74)	12 (20.69)		
Family history			0.053	0.818
Yes	45 (72.58)	41 (70.69)		
No	17 (27.42)	17 (29.31)		

### Inclusion and exclusion criteria

All patients confirmed as thyroid cancer by pathological diagnosis, and patients with Tg ≥10 ng/ml or recurrence or metastasis after operation were included. Patients in the experimental group were able to withstand radioactive iodine treatment or voluntary radioactive iodine treatment, and patients in the control group were either physically intolerant or refused radioactive iodine treatment. Patients having undergone radiotherapy and chemotherapy, and patients who are intolerant to surgery were excluded, patients with diseases such as respiratory, digestive and endocrine system excluded, patients with cognitive impairment and communication impairment excluded, and patients who did not cooperate with the examination excluded.

All receivers and their family members need to sign an informed consent form and cooperate with the medical staff to complete relevant medical treatment. The study was approved ethically by Ethics Committee of the university.

### Methods

All patients were treated with total thyroidectomy. The test group underwent radioactive iodine therapy on the 5th day after operation, 7 days for one course. The therapeutic dose was determined to be 80∼200 mCi according to the residual amount of thyroid tissues based on thyroid radionuclide results, with the drug of sodium iodide oral solution (purchased from Ansheng Kexing Pharmaceutical Co., Ltd., production batch number: SFDA approval number H20057721). The control group was treated with conventional anti-infection and prevention of complications. All patients were followed up for 36 months by telephone and WeChat after discharge.

### Observation indicators

The clinical treatment efficacy, quality of life, recurrent laryngeal nerve injury, postoperative metastasis and recurrence rate and survival rate within 3 years were compared between the two groups. Evaluation criteria for clinical treatment efficacy (11): clinical symptoms and signs of patients (enlarged thyroid asymmetry, nodular, with hard and fixed texture) disappears and returns to normal are markedly effective; those improve significantly, and their levels are close to normal levels are effective; those do not improve significantly or even worse is ineffective. The percentage of the sum of the number of markedly effective cases and that of effective cases in the total number of cases is the total effective rate. The European EORTC QLQ-C30 scale (12) was used to evaluate the postoperative quality of life of patients, including 12 items. They are 6 in symptom area (tiredness, nausea and vomiting, insomnia, loss of appetite, pain and diarrhea), 5 in function scale (cognition, emotion, society, body and role) and 1 in overall health status. The postoperative recurrent laryngeal nerve injury of patients includes unilateral and bilateral recurrent laryngeal nerve injury.

### Statistical methods

SPSS19.0 (Boyi Zhixun (Beijing) Information Technology Co., Ltd.) software was used to statistically process the data, GraphPad Prism 6 software to plot survival curve, χ^2^ test for the comparison of count data and rate. Measurement data were expressed as mean ± standard deviation (x̄±s), and compared using *t* test. When *P*<0.05, the difference is statistically significant.

## Results

### Comparison of clinical treatment efficacy between two groups of patients

In the test group, there were 40 people with markedly effective, 21 people with effective and 1 person with ineffective. The total effective rate was 98.39%. In the control group, there were 24 people with markedly effective, 18 people with effective and 16 people with ineffective. The total effective rate was 72.41%.

The total effective rate of the test group was significantly higher than that of the control group, with a statistically significant difference (*P*<0.05) (Table 2).

**Table 2:** Comparison and analysis of clinical treatment efficacy between two groups of patients [n(%)]

***Factors***	***Test group (n=62)***	***Control group (n=58)***	***X^2^***	***P***
Markedly effective	40 (64.52)	24 (41.38)	-	-
Effective	21 (33.87)	18 (31.03)	-	-
Ineffective	1 (1.61)	16 (27.59)	-	-
Total effective rate	61 (98.39)	42 (72.41)	16.63	<0.001

### Comparison of quality of life between two groups of patients

In the test group of patients, the fatigue score in the symptom area, the emotional function score of the function scale and the overall health status score were (13.57±13.39), (83.42±11.23) and (88.12±15.84), respectively, significantly better than those of the control group, which were (20.29±13.01), (73.23±5.36) and (68.08±12.38), respectively. The difference was statistically significant (*P*<0.05). There was no significant difference in the other scores of the quality of life (*P*>0.001) (Table 3).

**Table 3:** Comparison of EORTC QLQ-C30 score between two groups of patients

***Variable***	***Test group (n=62)***	***Control group (n=58)***	***t***	***P***
Symptom area				
Tiredness	13.57±13.39	20.29±13.01	2.785	<0.05
Nausea and vomiting	4.19±8.68	5.17±7.93	0.644	0.521
Insomnia	15.11±22.87	18.23±20.12	0.791	0.434
Loss of appetite	12.77±15.93	15.32±13.96	0.930	0.354
Pain	13.12±4.34	12.17±4.27	1.208	0.230
Diarrhea	2.54±7.35	2.18±7.87	0.259	0.796
Function scale				
Cognitive function	87.96±12.07	87.64±9.77	0.159	0.874
Emotional function	83.42±11.23	73.23±5.36	6.273	<0.05
Social function	87.56±15.09	87.72±13.36	0.061	0.091
Role function	94.72±8.75	93.42±7.93	0.851	0.397
Body function	91.37±7.93	89.95±8.31	0.958	0.340
Overall health status	88.12±15.84	68.08±12.38	7.6868	<0.05

### Comparison of recurrent laryngeal nerve injury between two groups of patients

In the test group of patients, there was 1 patient with unilateral permanent recurrent laryngeal nerve injury, 1 patient with unilateral temporary recurrent laryngeal nerve injury, none with bilateral permanent recurrent laryngeal nerve injury and 1 patient with bilateral temporary recurrent laryngeal nerve injury. In the control group of patients, there were 2 patients with unilateral permanent recurrent laryngeal nerve injury, 3 patients with unilateral temporary recurrent laryngeal nerve injury, none with bilateral permanent recurrent laryngeal nerve injury and 1 patient with bilateral temporary recurrent laryngeal nerve injury. There was no significant difference in recurrent laryngeal nerve injury between the two groups of patients (Table 4).

**Table 4:** Comparison of recurrent laryngeal nerve injury between two groups of patients [n(%)]

***Recurrent laryngeal nerve injury***	***Test group (n=62)***	***Control group (n=58)***	***X^2^***	***P***
Unilateral temporary injury	1 (1.61)	2 (3.45)	0.414	0.520
Unilateral permanent injury	1 (1.61)	3 (5.17)	1.178	0.278
Bilateral temporary injury	1 (1.61)	1 (1.72)	0.002	0.962
Bilateral permanent injury	0	0	-	-

### Comparison of metastasis and recurrence rate and survival rate within 3 years between two groups of patients

**Fig. 1:**
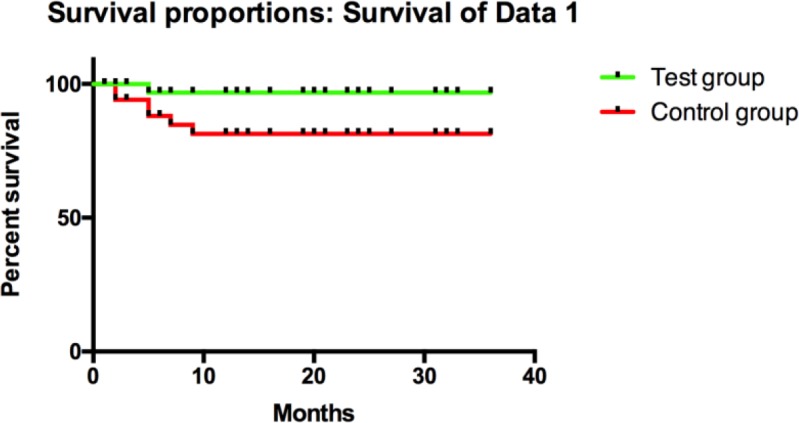
Comparison of 3-year survival rate between two groups of patients The 3-year survival rate of the test group was significantly higher than that of the control group (X^2^=3.947, *P*=0.047)

All patients were followed up for 36 months after discharge. In the test group of patients, there were 3 patients with metastasis and recurrence, and 1 patient died. The metastasis and recurrence rate was 4.8%, and the survival rate was 98.39%. In the control group of patients, there were 14 patients with metastasis and recurrence, and 6 patients died. The metastasis and recurrence rate was 24.14%, and the survival rate was 89.66%. The postoperative metastasis and recurrence rate of the test group of patients was lower than that of the control group (X^2^=9.179, *P*=0.002), but the survival rate of the test group was significantly higher than that of the control group (X^2^=3.947, *P*=0.047) (Table 5 and Fig. 1).

**Table 5:** Comparison of postoperative metastasis and recurrence rate and survival rate between two groups of patients [n(%)]

***Groups***	***Test group***	***Control group***	***X^2^***	***P***
Metastasis and recurrence rate	3 (4.80)	14 (24.14)	9.179	<0.05
Survival rate	61 (91.38)	52 (89.66)	3.947	<0.05

## Discussion

Thyroid cancer is a relatively common cancer in clinical practice, with a high incidence (13). Most of thyroid tumors originate in thyroid epithelial cells, only a few of which are metastasized and formed from other tumors (14). In general, as the preferred method of thyroid cancer, surgical treatment can basically control the occurrence and development of it (15). Although it is currently an effective way to treat thyroid cancer clinically, the effect of it alone on the clinical treatment efficacy and quality of life of thyroid cancer patients is not satisfactory (16). After total thyroidectomy, patients may have symptoms of thyroid function deterioration in addition to the metastasis and recurrence of tumor (17). Postoperative thyroid loss leads to the abnormality of the partial endocrine function of the body, patients more prone to adverse reactions such as poor emotional function, poor role function, easy fatigue and poor sleep. If not improved in time, it will cause serious consequences on the quality of life of patients. Radioactive iodine therapy can help the recovery of the thyroid function of patients, thus improving the quality of life of them (18). In order to improve the postoperative quality of life and clinical treatment efficacy of thyroid cancer patients, it has been clinically advocated for the treatment of thyroid cancer patients after total thyroidectomy combined with radioactive iodine therapy (19).

In this study, 120 thyroid cancer patients were divided into two groups. The test group was continuously treated with radioactive iodine after operation, the control group with conventional anti-infection.

The results showed that the two groups were compared in clinical treatment efficacy. The total effective rate was 72.41%. The total effective rate of the test group was significantly higher than that of the control group. The difference was statistically significant, indicating that the treatment efficacy of total thyroidectomy combined with radioactive iodine therapy is better than that of simple surgical treatment. The findings of Hong et al (20) also confirmed that the clinical treatment efficacy of radioactive iodine therapy after total thyroidectomy was better than that of surgical treatment alone.

The two groups of patients were compared in terms of the quality of life. According to our results, it indicates the use of radioactive iodine therapy plays an important role in improving the emotion and quality of life of patients. In another study (21), radioactive iodine therapy was beneficial to improve the emotion and quality of life of patients by improving their thyroid function, consistent with our conclusions.

Then, all patients were followed up for 36 months. The metastasis and recurrence rate of the test group was 4.8%, significantly lower than 24.14% of the control group. This suggests that total thyroidectomy combined with radioactive iodine therapy could effectively reduce the metastasis and recurrence rate and improve the survival rate of patients.

However, some patients will have symptoms of radiation thyroiditis or neck edema after operation (22). For them, glucocorticoids should be used in radioactive iodine therapy. In addition, the dose should be adjusted according to the actual situation. Nevertheless, in this study, there was no such case in patients, so it is hoped that attention can be paid to this problem by scholars in clinical research.

## Conclusion

The implementation of total thyroidectomy combined with radioactive iodine therapy in thyroid cancer can significantly improve the clinical treatment efficacy and quality of life of patients, worthy of further clinical promotion.

## Ethical considerations

Ethical issues (Including plagiarism, informed consent, misconduct, data fabrication and/or falsification, double publication and/or submission, redundancy, etc.) have been completely observed by the authors.
